# Extracellular Vesicles: A Comprehensive Review of Their Origins, Functions, and Therapeutic Potential

**DOI:** 10.3390/biomedicines14030495

**Published:** 2026-02-25

**Authors:** Madison B. Schank, Juan Zhao, Ling Wang, Jonathan P. Moorman, Zhi Q. Yao

**Affiliations:** 1 Center of Excellence in Inflammation, Infectious Disease and Immunity, James H. Quillen College of Medicine, East Tennessee State University, Johnson City, TN 37614, USA; zhaoj2@etsu.edu (J.Z.); wangl3@etsu.edu (L.W.); moorman@etsu.edu (J.P.M.); 2 Department of Internal Medicine, Division of Infectious, Inflammatory and Immunologic Diseases, Quillen College of Medicine, East Tennessee State University, Johnson City, TN 37614, USA; 3 Hepatitis (HCV/HBV/HIV) Program, James H. Quillen VA Medical Center, Department of Veterans Affairs, Johnson City, TN 37614, USA

**Keywords:** extracellular vesicles, exosome, biomarkers, EV isolation, drug delivery, immunotherapy

## Abstract

Extracellular vesicles (EVs) are membrane-bound particles secreted by most cell types that play a pivotal role in intercellular communication via transporting protein, nucleic acid, lipid, and metabolite cargos. Among EVs, exosomes are a well-characterized subtype, typically ranging from 10–150 nm in diameter and originating from the endosomal pathway via the formation of multivesicular bodies that fuse with the plasma membrane. EVs/exosomes can be isolated from various biological fluids and cultured cells, with production and yield influenced by the cell type and culture conditions. Isolation methods, including ultracentrifugation or density-based ultracentrifugation, tangential flow filtration, size-exclusion chromatography, immunoaffinity and membrane-affinity capture, and recently developed commercial equipment, offer distinct advantages and limitations in terms of purity, scalability, and exosome integrity. Characterization techniques, such as nanoparticle tracking analysis (NTA), transmission electron microscopy (TEM), cryogenic electron microscopy (cryo-EM), atomic force microscopy (AFM), Western blotting, flow cytometry, and dynamic light scattering (DLS), assess exosome size, morphology, and biomarker expression. Given their biocompatibility and inherent targeting capabilities across a diverse range of diseases, EVs/exosomes hold clinical promise as diagnostic biomarkers, cell-free therapeutics, drug delivery vehicles, immune modulators, and in regenerative medicine. However, these emerging fields in exosome medicine continue to face challenges in standardizing EV sourcing, production, purification, yield, bio-targeting, drug loading, and drug delivery. While EVs/exosomes represent a rapidly advancing frontier in biomedical science, robust protocols for standardization and scalable production will be essential for their successful translation into clinical applications. This article provides a comprehensive overview of EV/exosome origins, their biological functions, the approaches for their isolation and characterization, and their therapeutic potential.

## 1. Introduction

**Figure 1:**
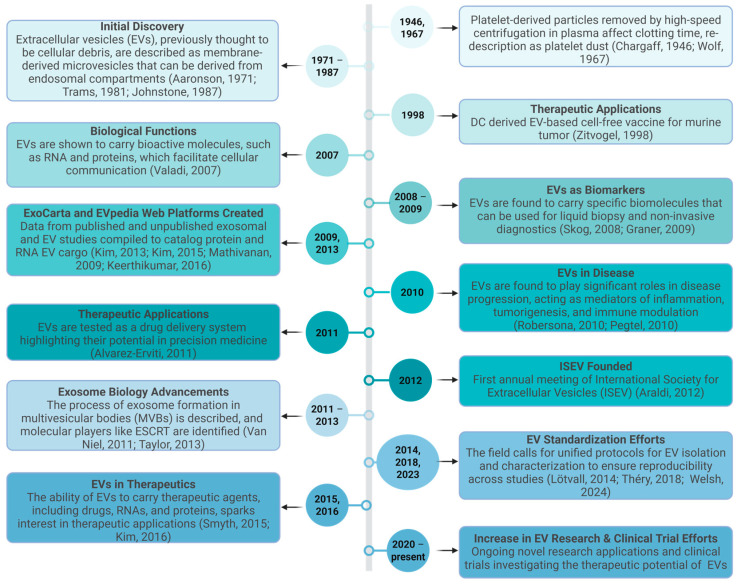
A historical timeline of EV research [1,2,3,4,5,9,10,11,12,13,14,15,16,17,18,19,20,21,22,23,24,25,27,31]. Created in BioRender. Schank, M. (2026) https://biorender.com/fy6q27p (accessed on 18 February 2026).

The first evidence of extracellular vesicles (EVs) originated in 1947, during a study highlighting that high-speed centrifugation of plasma resulted in the isolation of a small pellet and altered plasma clotting time, implicating the presence of a clotting factor in the isolated pellet [1]. A subsequent study defined the isolated pellet as “platelet-dust” and identified that these particulates were released from intact platelets and enriched in lipid content [2]. Later studies further characterized unique vesicle-like structures with a small diameter of 40 nm by electron microscopy (EM) [3,4]. During research throughout the 1980s, studies described vesicles released during reticulocyte maturation that originated from multivesicular bodies (MVBs), introducing the term “exosome” to denote vesicles of endosomal origin [5,6]. These vesicles were often considered to be cellular waste products; however, later work demonstrated that a subset of EVs is generated via the endosomal pathway and participates in intercellular communication [7,8]. As characterization techniques advanced, it became evident that EVs represent a heterogeneous population of membrane-bound particles differing in size, composition, and biogenesis. Consequently, terminology in the field has evolved, and the International Society for Extracellular Vesicles (ISEV) developed the Minimal Information for Studies of Extracellular Vesicles (MISEV) guidelines to standardize nomenclature and reporting practices. The current guidelines recommend the use of the umbrella term EVs unless the specific biogenesis pathway has been experimentally demonstrated [9,10,11]. Growing evidence has revealed a significant biological and therapeutic role for EVs [12,13,14,15,16,17,18,19,20,21,22,23]. This is especially true as their applications as cell-derived therapeutics for various clinical scenarios have only recently been recognized [9,24,25,26,27,28,29,30,31]. Figure 1 highlights a timeline of advances in the field of EV research.

## 2. EV Nomenclature, Biogenesis, Characteristics, and Function

### 2.1. EV Nomenclature

MISEV guidelines have recommendations regarding the definition, nomenclature, isolation, characterization, markers, functional studies, and experimental reporting of EV-related research. The current MISEV guidelines define EVs as cellular released lipid bilayer-containing particles that cannot replicate on their own [10]. Subsets of EVs exist depending on their biogenesis mechanism. These include exosomes (originating from the endosomal system), ectosomes/microvesicles (originating from the plasma membrane), and apoptotic bodies (produced during apoptosis); however, unless the subcellular origin is experimentally demonstrated, MISEV discourages using these terms and recommends the broader EV nomenclature.

Cells also produce non-vesicular extracellular particles (NVEPs), which do not have a lipid bilayer and are produced in much higher rates than EVs in biological matrices. EVs may also be produced during cellular processes, including migration (migrasomes) or programmed cell death (apoptotic bodies). MISEV also recommends cautious use of “operational term” prefixes denoting when EVs are separated on the basis of specific characteristics, such as size, density, molecular composition, or cellular origin. These include small EVs (<200 nm in diameter) and large EVs (>200 nm). According to MISEV recommendations, categorizing EVs as small or large EVs, exosomes, or ectosomes is challenging. Most isolation and characterization methods do not provide sufficient specificity to confidently assign EVs to these subtypes, and therefore, such terminology should be used cautiously.

### 2.2. EV Biogenesis

**Figure 2:**
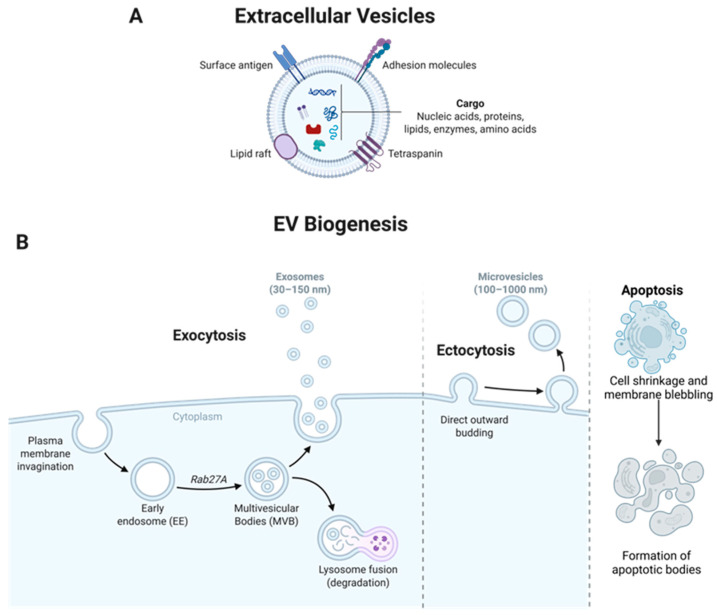
EV structure and biogenesis pathways. (**A**) EV cargo. EVs contain tetraspanin proteins and encapsulate amino acids, RNA, DNA, proteins, and enzymes that allow for essential intercellular communication. (**B**) EV biogenesis. Exosomes are formed via the process of exocytosis through the endosomal pathway, where plasma membrane invagination forms a cup-shaped structure containing cell surface proteins and extracellular components. Early endosomes form and mature into multivesicular bodies (MVBs), encapsulating intraluminal vesicles (ILVs), including exosomes. MVBs can then either fuse with the plasma membrane, resulting in the release of exosomes to facilitate intercellular communication, or with the lysosome to form endolysosomes. Microvesicles/ectosomes are formed via direct outward budding of the plasma membrane. Apoptotic bodies are formed during programmed cell death were membrane blebbing results in release of apoptotic bodies. Created in BioRender. Schank, M. (2026) https://biorender.com/w10fnsp (accessed on 11 February 2026).

Studies have indicated that EV production and release are conserved across nearly all cell types and in distinct life forms, including prokaryotes, eukaryotes, bacteria, archaea, fungi, and parasites [10,32]. EVs are a component of various biological fluids such as blood, plasma, urine, saliva, breast milk, amniotic fluid, lymph, and cerebrospinal fluid, implicating their diverse functions throughout the human body [33,34]. EVs contain a variety of biomolecules, including proteins, lipids, RNA (including microRNA, mRNA, and other non-coding RNAs), and metabolites (Figure 2A) [25,26,35,36,37,38,39,40]. EV contents reflect the cellular state of the parent cell and, as a result, EVs have become increasingly recognized as important players in various biological processes, including intercellular communication, signal transduction, immune modulation, and tumor progression and metastasis [41]. Specifically, EVs transfer molecular cargos (both membrane and luminal localized) to recipient cells to regulate gene expression and function, contributing to intercellular signaling and tissue homeostasis. Furthermore, EVs can function similarly to antigen-presenting cells (APCs) by presenting antigenic epitopes to immune cells, thereby facilitating immune responses through the expression of Major Histocompatibility Complex (MHC) class I and II molecules [42]. The inherent characteristics, conserved nature, and diversity of EVs highlight the ability to optimize and engineer EVs for a wide range of clinical applications, and thus, EVs have an integral and emerging role in the human body and in medicine.

#### 2.2.1. Exosomes

Exosomes are live-cell-produced small EVs, typically ranging from 30–150 nm in diameter [43]. Exosomes are generated via the endosomal pathway, beginning with the inward budding of the plasma membrane to form early endosomes containing cell surface proteins and extracellular components. These structures enter the cell cytoplasm to become early endosomes. Early endosomes then develop into multivesicular bodies (MVBs), which enclose intraluminal vesicles (ILVs), including exosomes. MVB and ILV biogenesis is typically facilitated by the endosomal sorting complex required for transport (ESCRT) pathway, which involves approximately 30 proteins that form five distinct protein complexes, including ESCRT-0, -I, -II, -III, and AAA ATPase Vps4. This process is ATP-dependent and also relies on a series of accessory proteins, such as the ALG-2-interacting protein X (ALIX) homodimer and tumor susceptibility gene 101 (TSG101), which help to coordinate the sorting and budding of ILVs [26,44]. Ras-related proteins in brain (Rab) proteins, particularly Rab27a and Rab27b, are also involved in regulating intracellular membrane trafficking and fusion with MVBs [45]. MVB and ILV biogenesis can also be completed in ESCRT-independent mechanisms involving lipid-driven membrane budding and tetraspanin-enriched microdomains [25,46]. Following MVB biogenesis, MVBs can either fuse with the plasma membrane, leading to the release of exosomes into the extracellular space for intercellular communication, or the lysosome for degradation (Figure 2B).

#### 2.2.2. Ectosomes/Microvesicles and Apoptotic Bodies

Microvesicles, also referred to as ectosomes, are generated through the outward budding and fission of the plasma membrane. They are typically heterogeneous and irregular in morphology, with diameters generally ranging from approximately 100–1000 nm. They also commonly express selectins, ARF6, CD40, cytoskeletal proteins, heat shock proteins, and integrins (Figure 2B). Apoptotic bodies are produced during programmed cell death and are formed through membrane blebbing and cellular fragmentation and typically contain nuclear components and histones. These vesicles are larger and more variable in size, typically exceeding 1000 nm in diameter (Figure 2B) [47].

#### 2.2.3. Bacterial EVs

Bacterial EVs are produced by both Gram-negative and Gram-positive bacteria through distinct biogenesis mechanisms. In Gram-negative bacteria, vesicles originate primarily from the outer membrane and are commonly referred to as outer membrane vesicles (OMVs). These can form through membrane blebbing or explosive cell lysis, and may also incorporate periplasmic and inner membrane components [48]. Gram-positive bacteria generate vesicles derived from the cytoplasmic membrane that traverse the peptidoglycan layer. Thus, molecular markers of bacterial EVs reflect their membrane origin and bacterial classification. Gram-negative EVs are typically enriched in lipopolysaccharide (LPS) and outer membrane proteins (e.g., OmpA), whereas Gram-positive EVs commonly contain lipoteichoic acid (LTA) and cytoplasmic membrane-associated proteins [10,32,49,50,51].

### 2.3. EV Characteristics

EV cargos are incorporated into both the exosome surface and lumen during their production. The mechanism by which exosomes are formed plays a critical role in the process of cargo sorting, and thus, differential identification of EVs as exosomes, microvesicles, and apoptotic bodies can be completed using specific markers unique to each EV subset [47]. EV cargo sorting is a highly selective, active, and regulated process that is influenced by cell type, physiological state, and external stimuli, which has direct influences on target cell specificity, immune modulation, and biomarker utilization, etc. [52,53]. ESCRT-dependent mechanisms primarily recognize and package ubiquitinated proteins and internalized receptors, while ESCRT-independent mechanisms are tightly regulated by ceramide, tetraspanins, and lipid microdomains [26,54,55]. EV surface cargo includes tetraspanins CD63, CD9, and CD81, which are derived from the plasma membrane and endosomes of the host cell and also participate in endosomal sorting during exosome biogenesis [35,36]. Additionally, receptors, integrins, and lipids are incorporated into the EV membrane. The luminal cargo of EV includes a diverse array of molecules, such as amino acids, enzymes, proteins, and various types of RNA, including mRNA, lncRNA, and miRNA. Currently, 13,476 proteins, 3408 mRNAs, and 10,755 miRNAs have been identified from isolated exosomes according to the ExoCarta database [19]. These studies confirm that the cell source of exosomes dictates the specific cargos encapsulated in exosomes. However, approximately 80% of the proteins in exosomes are highly conserved across different cell types. Commonly incorporated proteins include HSP60, 70, and 90, as well as ALIX, TSG101, and clathrin (Figure 2A). These conserved proteins are commonly used as biomarkers for exosomes [25,26,37,38,39,40].

EVs have also been reported to associate with genomic and mitochondrial DNA; however, the abundance, localization, and functional relevance of DNA within EVs remain controversial and highly dependent on isolation and characterization methods [40,56,57,58]. Because reported DNA association with EVs is highly dependent on isolation and analytical methods, inclusion of appropriate controls, such as nuclease treatment, detergent disruption, and assessment of EV-depleted fractions, is critical to support vesicle-associated localization.

### 2.4. EV Functions Following Release

Following EV release into the extracellular space, EVs interact with target cells by various mechanisms, including plasma membrane fusion and cargo release, endocytosis, and surface receptor interactions. The specific mechanisms by which EVs interact with a target cell determine the fate of the internal cargos. In the case of plasma membrane fusion, the EVs fuse directly with the target cell membrane, immediately releasing their cargos into the cell cytoplasm. This mechanism represents a direct and efficient form of cargo delivery. Alternatively, endocytosis involves EV engulfment by the target cell membrane and internalization into an endosome. This process releases the EV cargos to be processed within the cell, often through endosomal pathways. If the EV cargos are not released into the cytoplasm, they can be degraded through the lysosomal pathway as part of the cell’s normal waste management processes [41,59,60]. Surface receptor interactions involve the binding of molecules on the EV surface, such as tetraspanins, integrins, and heat shock proteins, with complementary receptors on the target cell surface. For example, tetraspanins (e.g., CD9, CD63, and CD81), which are commonly used as exosomal markers) and integrins can interact with cell-specific receptors, promoting binding and internalization of the exosomes. This process often leads to endocytosis, where the EVs are engulfed by the target cell membrane and internalized into endosomes. Once inside the cell, the EV cargos can be processed and delivered to the cytoplasm, allowing the EV to exert their effects on the target cell.

Recent studies have investigated the biodistribution of EVs in animals, either by in situ analysis of whole live animals or ex vivo analysis of harvested organs. Typically, EVs primarily localize to the liver with peak detection at 1 h post-treatment and stable levels for prolonged periods (24–72 h) [61,62,63,64]. Albeit at lower levels than liver localization, EVs have also been shown to consistently localize in the lungs, spleen, kidneys, GI tract, brain, heart, bladder, skeletal muscle, bone/bone marrow; however, the distribution of EVs in these anatomical locations is optimal at middle (2–12 h) and late time-points (≥24 h) [61,62,65,66,67]. Potential causes for the significant liver accumulation of EVs could be attributed to certain features of the liver, including large size, high blood flow, the specialized leaky capillaries of liver sinusoids, and its role in blood detoxification, as well as the presence of certain liver cell receptors compatible with EV integrin expression [68,69].

## 3. EV Sourcing, Production, and Isolation

### 3.1. EV Sourcing and Production

Cell culture media from in vitro cell cultures is a commonly used (83%) source of EVs and is the leading material used for EV collection [70]. However, certain modifications are necessary to properly collect cell-derived EVs. A key consideration is that most cell lines are cultured in media supplemented with fetal bovine serum (FBS), which contains high levels of cow-derived EVs. These FBS-oriented EVs can contaminate the purified EVs and interfere with their downstream applications [71]. This is especially important when the EV source is critical for the intended application. For therapeutic applications, EVs are typically isolated from cell sources that are compatible with the target cells. To minimize contamination from FBS-derived EVs, it is essential to deplete FBS EVs from serum before culturing the cells. This can be achieved through ultracentrifugation or by using commercially available EV-depleted FBS. Alternatively, some researchers (37%) opt for exclusively serum-free culture conditions [70]. Since EVs are cell-derived, their cellular origin significantly influences both the cargo encapsulated within the EV membrane and the proteins expressed on their surface. Mesenchymal stem cells (MSCs) are often used as a cellular source of EVs due to their large exosome production levels, low immunogenicity, and high biocompatibility. Given these beneficial features, MSC-derived EVs have been used for clinical applications [72].

To increase the yield and scale up production, bioreactors can be used to optimize high EV yield by providing a controlled environment under specific culture conditions (including pH, temperature, nutrient availability, and oxygen supply), allowing cells to expand optimally and produce a high yield of cellular products [73,74,75,76]. For example, the hollow fiber cell culture system more closely approximates the mammalian circulatory system to better mimic in vivo conditions. Hollow fibers are small tube-like filters approximately 200 microns in diameter. Cells are grown in the extracapillary space (space on the outside of fibers). This allows for a high surface area to volume ratio (for example, 3000 square cm of area in a volume of 20 mL), which allows for the continuous culture of cells at high densities (such as 1 × 10^8^/mL or higher) for extended periods. Cells are provided continuous flow of media for the provision of nutrients and oxygenation by a fiber cell system dual pump. The product can be harvested, and glucose levels (as an indicator of cell numbers and growth levels) can be checked in the reservoir bottle. Data have shown a 100-fold increase in EV yield utilizing the fiber cell culture system [77].

### 3.2. EV Isolation Techniques

**Figure 3:**
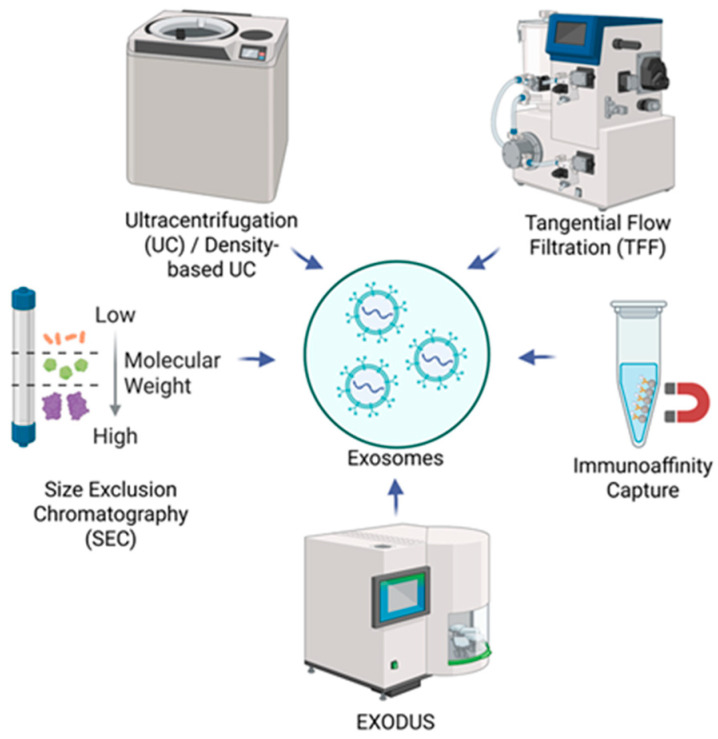
EV isolation techniques. The primary isolation techniques typically used include ultracentrifugation (UC), density-based UC, tangential flow filtration (TFF), size exclusion chromatography (SEC), immunoaffinity capture, and newly developed commercial isolation instrumentation (EXODUS) that separates particles on the basis of density, size, shape, protein/receptor expression, and solubility-based precipitation. Created in BioRender. Schank, M. (2026) https://biorender.com/qqspvh7 (accessed on 23 January 2026).

Following the production of EV starting material, EVs need to be isolated, characterized, and quantified for later applications. Various techniques have been established to isolate EVs based on several variables, including density, size, shape, protein/receptor expression, and solubility-based precipitation. Correspondingly, the overall purity, yield, sample requirements, and timeframe vary based on the isolation method used. The primary isolation techniques currently used include ultracentrifugation (UC), density-based UC, tangential flow filtration (TFF), size exclusion chromatography (SEC), immunoaffinity capture, and newly developed commercial isolation instrumentation (Figure 3) [78,79]. A summary of the characteristics and advantages of these isolation methods is shown in Table 1. A variety of other techniques have been used for EV isolation throughout history; however, they each have varying degrees of yield, purity, required starting material volume, scalability, etc. These approaches and their typical use across EV researchers have been reviewed in more detail previously [10,43,70].

**Figure 4:**
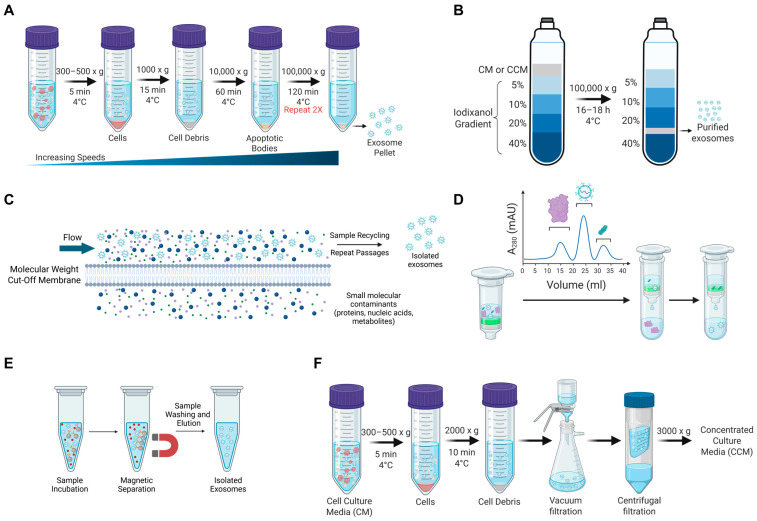
A schematic overview of EV isolation and concentration approaches. (**A**) Stepwise protocol of differential ultracentrifugation to separate particles based on their size and density via increasing centrifugal force. (**B**) Schematic of density-based centrifugation for the isolation of EVs using a density gradient of iodixanol and ultracentrifugation. (**C**) Graphical representation of tangential flow filtration (TFF) for EV isolation based on particle size. (**D**) Summary of size exclusion chromatography (SEC) principle for EV isolation. (**E**) Schematic of magnetic bead-based immunoaffinity capture for EV isolation. (**F**) Stepwise protocol for concentration of EV cell culture media for further use in EV isolation. Created in BioRender. Schank, M. (2026) https://biorender.com/g14f6wn (accessed on 11 February 2026).

The initial method used for the isolation of EVs was differential ultracentrifugation, during which particles of differing size and density are depleted from a sample following a stepwise increase in centrifugal force. Multiple variations of this protocol have been used, but the general protocol is as follows: 300–800× *g* for 5 min, 1000× *g* for 15 min, 10,000× *g* for 1 h, and 100,000× *g* for 2 h twice, with all steps performed at 4 °C (Figure 4A) [10]. This process depletes cellular debris and contaminants, organelles, and large EVs present in the cell culture media stepwise. Ultracentrifugation, including differential centrifugation, is a leading isolation approach (used by more than 80% of EV investigators) [70,80,81]. For the isolation of EVs, however, this approach is limited by the need for a large starting volume of EV source (i.e., cell culture media), the potential damage of EVs, relatively low to moderate purity, and low reproducibility and sample throughput (Table 1) [78].

Alternatively, density gradient centrifugation is also used to purify EVs based on their size and density, using a density-forming material such as iodixanol (OptiPrep) or sucrose, and is typically used by 20% of EV researchers [70]. This process separates particles by typically creating a density gradient of iodixanol (ranging from 5–40%) and layering cell culture media (CM) above this gradient. The sample is centrifuged at 100,000× *g* for 16–18 h at 4 °C, allowing particles to separate and settle at their corresponding density (Figure 4B). EVs will typically be collected between the 20 and 40% iodixanol solutions. This process has high purity with generally low levels of contaminants; however, this isolation method is low throughput, and the need for the density gradient slightly increases the cost per sample (Table 1) [82].

Tangential flow filtration (TFF), also referred to as cross-flow filtration, is a filter-based EV isolation approach based on particle size (used by ~18% of EV researchers) [70]. Typically, starting material is pre-clarified by centrifugation or low-speed filtration to remove cells and cell debris. TFF allows samples to flow tangentially across the surface of the filter membrane, as opposed to dead-end filtration, to minimize clogging and continuous processing of large volumes of starting material. TFF utilizes a semi-permeable membrane with a defined molecular weight cut-off (MWCO), usually between 100 and 500 kDa, which allows for EVs to be retained, while also removing smaller molecule contaminants, including proteins, nucleic acids, and metabolites [83]. A parallel flow from a pump allows liquid and molecules smaller than the membrane pores to pass through the filter and become the permeate perpendicularly to the flow applied, while larger particles are retained in the fluid (Figure 4C) [84]. The sample is repeatedly passed over the membrane while additional buffer is added. The advantage of TFF is that the continuous flow and recycling of the fluid increases the number of passages across the filter, improving the removal of contaminants, while also enabling higher-throughput processing in a relatively short time [10]. TFF is also beneficial compared to centrifugation approaches as the EVs have undergone no centrifugal force, promoting the recovery of intact EVs with little structural damage; however, the choice of MWCO membrane, pressure, and flow rate are critical variables in ensuring intact EVs with high purity (Table 1).

Size-exclusion chromatography (SEC) is another technique commonly used (~15% of EV researchers) for EV isolation [70]. SEC is a gentle and reliable method for EV isolation based on particle size. Specifically, SEC separates particles based on their hydrodynamic radius. Samples are typically pre-clarified through centrifugation or TFF. A column is packed with porous beads, and as the sample passes through, particles will be eluted based on their size. Larger particles (larger than the matrix pore size) are unable to pass through the matrix pores and will be collected as early fractions. Conversely, smaller particles will penetrate the porous stationary phase, slowing down their movement and causing them to elute in later fractions (Figure 4D) [10,43]. Benefits of SEC include low shear stress to preserve EV integrity and high purity due to removal of proteins and small contaminants; however, application of SEC for EV isolation is limited by low specificity and moderate yield (Table 1). Also, EVs can be isolated using commercially available kits. For example, the Qiagen exoEasy Maxi kit, which uses a membrane-based affinity binding to isolate exosomes and other EVs from serum and plasma or cell culture supernatants, can be used for EV isolation. However, studies have suggested that while this kit produces high total RNA yield from EVs, the method co-isolates plasma proteins and does not produce high levels of EV-associated proteins, indicating low purity and yield [85,86]. The overall specificity and yield using these methods for isolation of EVs are variable, depending on the quality and features of SEC and kit columns from different commercial companies.

Immunoaffinity capture is an EV isolation approach utilizing specific binding between antibodies or affinity ligands and exosomal markers on the EV surface, such as CD9, CD63, CD81, ALIX, etc. The antibodies/ligands are immobilized by a variety of supports, including magnetic beads, microfluidic devices, or microtiter plates. For example, when utilizing magnetic bead-based capture, samples are allowed to incubate with the antibody-coated magnetic beads (Figure 4E). Following an incubation period, exosomal proteins are bound to antibodies, a magnetic field is applied, the unbound sample containing contaminants such as proteins and nucleic acids is removed by washing, and the captured EVs can be eluted for collection [79,87]. Advantages of immunoaffinity capture include high purity and specificity and low stress to EV structure; however, it generally produces a low to moderate EV yield with high cost (Table 1).

Recently, novel approaches for EV/exosome isolation have been developed to overcome many of the challenges traditional isolation methods face, including low levels of sample processing speed, yield, and purity, all of which have limited exosome application in clinical settings. In 2021, a group developed Exosome Detection via the Ultrafast-Isolation System (EXODUS) technology. EXODUS is an automated, label-free platform that utilizes negative pressure oscillation (NPO) and a double-coupled harmonic oscillator to induce membrane vibrations [88]. These dual-frequency transverse waves enhance the purification efficiency of exosomes by removing free nucleic acids and proteins, allowing for faster, higher-yield, and purer isolation compared to conventional techniques. Following the removal of contaminants, EVs/exosomes are captured by a nanoporous membrane. Since its development, the EXODUS has been used by multiple investigators and has shown improved exosome metabolic viability [89,90].

Across all EV isolation strategies, common confounders include co-isolated soluble proteins and nucleic acids, lipoproteins, protein aggregates, and other non-vesicular nanoparticles, which can complicate the interpretation of downstream analyses [10,40,91]. Importantly, these contaminants may not only be present in EV preparation, but they may also absorb onto the vesicle membrane, contributing to or modifying the EV surface biomolecular corona upon release into biological fluids. The stable hard layer and dynamic soft layer significantly influence EV biological identity, mobility, cellular uptake, and in vivo distribution [92,93,94]. Differential ultracentrifugation and density gradient approaches reduce some soluble contaminants (but co-isolate NVEPs at high speeds), but may co-pellet protein aggregates or lipoproteins that associate with the vesicle surface, potentially generating an enriched corona. High centrifugal forces may also compromise vesicle integrity, altering membrane topology and surface protein presentation. SEC improves the separation of vesicles from soluble proteins but may allow the co-elution of similarly sized NVEPs and often trades purity for yield. TFF enables scalable processing but can enrich non-EV nanoparticles depending on membrane cutoffs and flow conditions. Immunoaffinity-based methods increase specificity for defined EV subpopulations but may bias recovery toward selected markers, mask native surface epitopes, or displace physiologically associated corona proteins, while also limiting scalability. Emerging microfluidic approaches such as EXODUS aim to balance yield, purity, and processing time, yet remain subject to size-based co-isolation of NVEPs. These method-dependent confounders underscore the need to interpret EV data in light of isolation-specific trade-offs among yield, purity, scalability, and vesicle integrity.

Furthermore, it has previously been reported that at least 59% of EV researchers use a combination of isolation techniques [70]. This combination approach can enhance purity and specificity, which may be essential when using the produced EVs in a GMP-compliant manner or for in vivo applications. The Extracellular RNA Communication Consortium (ERCC) is an NIH Common Fund program focused on extracellular RNA (exRNA) biology and technologies that identify, characterize, and isolate exRNA carriers, such as extracellular vesicles. ERCC (https://exrna.org/resources/ercc2-tech-query/ accessed on 22 January 2026) provides a resource for exploring various techniques related to extracellular RNA (exRNA) research. It allows users to query methods related to particle separation, such as microfluidics, filtration, and UC, as well as single EV analysis methods like flow cytometry, microscopy, and nanoparticle tracking analysis (NTA). The page is designed to help researchers select appropriate methods for their specific exRNA-related studies. The ideal EV isolation approach will vary based on a series of variables, including starting material, desired yield, batch-to-batch consistency, etc.; however, the wide range of isolation techniques and new resources highlighting their strengths and limitations provide a range of options that can be further optimized for specific experimental and application requirements.

Overall yield and purity for EV isolation approaches can be further improved by a series of centrifugation and concentration steps to produce concentrated culture media (CCM), which can then be further processed for EV isolation. For example, culture media is centrifuged at 300–500× *g* for 5 min at 4 °C to remove intact cells. The supernatant is further centrifuged at 2000× *g* for 10 min at 4 °C to pellet cellular debris. The supernatant can then be filtered using a vacuum filter with a 0.1 µm membrane, followed by concentration to ~1.5 mL with a centrifugal filter device (100 K nominal molecular weight limit (NMWL) filter) (Figure 4F) [71]. Typically, large volumes of CM are required for generating appropriate EV yields. Given the limitation in volume capacity and time required to process high-throughput samples, CCM preparation can be used to overcome this limitation and concentrate the EV sample before downstream isolation techniques.

## 4. EV Characterization

Following EV isolation, it is essential to thoroughly characterize the sample. This typically involves assessing parameters such as morphology, size, concentration, surface charge, density, biomarker expression, yield, and purity [87]. EV characterization techniques can be broadly classified into bulk (population level) and single-vesicle approaches (outlined in Table 2). Bulk approaches assess average properties across the entire EV population to provide information on size distribution, concentration, and overall cargo content. These include Western blotting, dynamic light scattering (DLS), nanoparticle tracking analysis (NTA, bulk mode and fluorescence mode), and mass spectrometry-based proteomics. Single-vesicle approaches provide insights into heterogeneity by analyzing individual EVs to allow for direct visualization of morphology, size, and surface marker composition of individual EVs. Techniques in this category include electron microscopy (EM), such as scanning EM (SEM), transmission EM (TEM), and cryogenic (cryo-EM), atomic force microscopy (AFM), and high-resolution flow cytometry (FCM) [43]. Together, bulk and single analysis methods provide a complementary and comprehensive assessment of EV quality, composition, and heterogeneity.

Microscopy was among the earliest methods used to characterize EVs and remains essential for assessing their morphology and structural features with high spatial resolution, often approaching 1 nm. Transmission electron microscopy (TEM) involves negative staining and dehydration of EV samples before imaging, allowing visualization of membrane structure and size. However, the dehydration and staining steps can induce artifacts, potentially altering EV morphology [79,95]. Scanning electron microscopy (SEM), which also involves dehydration and negative staining, incorporates sputter-coating with a conductive material to enhance image quality and enables three-dimensional visualization. SEM has been reported to better preserve the spherical shape of EVs compared to TEM [87].

Cryogenic electron microscopy (cryo-EM), a variant of TEM, overcomes the dehydration artifacts by maintaining samples in a hydrated, vitrified state. EV suspensions are applied to a grid, rapidly frozen in liquid ethane, and stored in liquid nitrogen, enabling imaging of EVs in their native morphology without staining [96]. Atomic force microscopy (AFM) offers a complementary approach by scanning EVs immobilized on a mica surface (which can be functionalized with antibodies) using a silicon probe. This technique provides nanometer-scale topographical information and preserves EV shape during imaging, though sample dehydration may still introduce some distortion [97]. While microscopy-based methods are highly effective for analyzing size, shape, and structural integrity, they are generally complicated in procedure and costly, and not suitable for quantitative assessment of EV concentration.

Western blotting is a widely used method for EV/exosome characterization, primarily to detect conventional exosomal markers such as CD9, CD63, CD81, ALIX, and TSG101. While effective for confirming the presence of these proteins, Western blotting does not provide information on particle size, morphology, or overall purity, and no single protein marker has been definitively established to confirm exosome purity [19,87]. To address these limitations, bead-based flow cytometry has been adapted for semi-quantitative analysis by labeling exosomal surface markers. However, traditional flow cytometers often lack the sensitivity to reliably detect exosomes due to their small size, typically below 200 nm. To overcome this, more advanced technologies such as imaging flow cytometry (IFCM) and nano-flow cytometry (nanoFCM) have been developed. These platforms offer improved resolution and sensitivity, enabling more accurate characterization of exosome populations, including size distribution, surface marker profiling, and particle concentration [98]. Furthermore, a novel method termed direct stochastic optical reconstruction microscopy (dSTORM) has been developed for mapping the surface and internal spatial distribution of EV/exosome proteins. dSTORM is a form of high-resolution single-molecule localization microscopy that relies on photoswitchable fluorophores that stochastically blink on and off, allowing individual molecules to be localized with high precision over time to reconstruct a super-resolved image. While this approach allows for a visualization of EVs and potentially visualization of cargo distribution within EVs, it is not suited for bulk characterization of a sample and does not give insights into size and concentration [99].

DLS and NTA utilize similar principles for EV particle characterization. Both approaches measure the Brownian motion of particles and utilize the Stokes–Einstein equation to assess their morphological size; however, NTA has become a leading technique used for EV characterization due to its ability to not only visualize EV light scattering for size estimation, but also characterize concentration [43,100]. During NTA, the EV sample is applied to a microscope camera capable of tracking the Brownian motion. NTA has higher resolution and is typically suitable for particles ranging from 70–300 nm, while DLS is better suited for particles in the 1 nm to several µm range. Additionally, DLS has a limited application to heterogeneous mixtures, as the intensity of scattered light is more sensitive to detecting larger particles in suspension. Because of its ability to simultaneously measure size distribution and particle concentration, NTA is widely used for EV characterization, particularly for heterogeneous populations. More recently, fluorescence-based nanoparticle tracking analysis (NTA) has been developed, enabling measurement of EV concentration and size in conventional scatter mode, as well as detection of fluorescently labeled EVs [101,102]. Vesicles may be labeled using lipophilic dyes to identify membrane-bound particles, nucleic acid-specific dyes to indicate DNA or RNA cargo, or fluorescent antibodies targeting specific EV-associated markers. Assessment of fluorescence signals and their co-localization allows estimation of EV subpopulations and their relative abundance within the total particle population, increasing specificity and sensitivity. Due to the wide range of characterization parameters that different techniques can assess, including size, concentration, morphology, and molecular content, EVs are most commonly analyzed using a combination of methods to ensure a comprehensive evaluation of their properties.

## 5. EV Therapeutic Applications

EV research gained significant momentum following a pivotal 1998 study demonstrating that dendritic cell (DC)-derived EVs could stimulate anti-tumor immune responses in mice by presenting peptide-loaded MHC class I and II molecules, along with T cell co-stimulatory markers such as CD82 and CD63 [12]. This study provided the first in vivo evidence of EVs functioning as therapeutic agents and laid the foundation for subsequent investigations into their clinical potential. Early follow-up studies focused primarily on the therapeutic promise of MSC-derived EVs in medicine, especially in tissue regeneration, tumor inhibition, and immune modulation. It has since become clear that many therapeutic effects are originally attributed to MSCs and mediated through EV-driven intercellular communication. In many cases, these effects can be replicated by direct application of MSC-derived EVs, underscoring their utility as a cell-free therapeutic alternative [103].

Today, EVs are under very active investigations for a wide range of medical conditions due to their ability to transfer molecular cargos (including proteins, RNAs, lipids, and drugs) between cells, modulate immune responses, and influence target cell behavior. Their applications span oncology (e.g., immunotherapy, drug delivery, diagnostics), neurodegenerative disorders (such as Alzheimer’s, Parkinson’s, and multiple sclerosis), cardiovascular diseases, diabetes, osteoarthritis, alopecia, preeclampsia, infectious diseases (including long COVID), autoimmune disorders (e.g., rheumatoid arthritis and lupus), wound healing, organ transplantation, and gene therapy. As of 12 February 2026, using the terms “extracellular vesicles” or “exosomes,” a total of 826 clinical trials were identified with no duplicates. Among these, 479 were classified as interventional studies, 343 as observational studies, and 4 as expanded access studies, with the majority in early-phase trials (Phases I–II) (www.clinicaltrials.gov, accessed on 12 February 2026).

**Figure 5:**
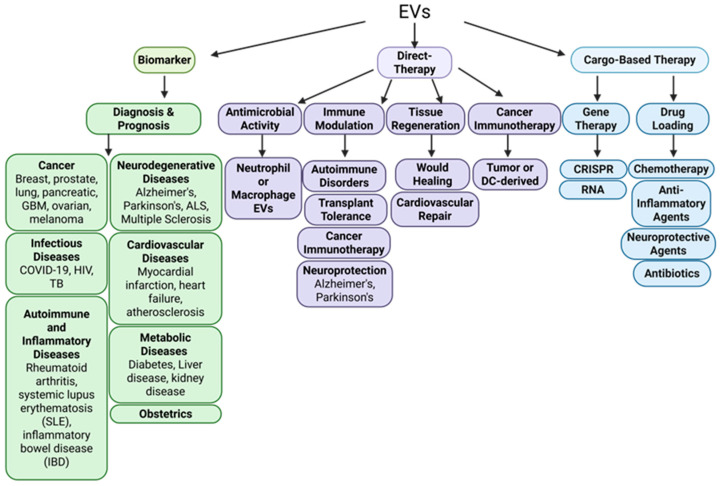
Diagram of therapeutic applications of EVs. EVs have therapeutic potential across three major applications: as a biomarker, direct therapy, and cargo-based therapy. Each of these uses has a diverse range of potential clinical applications and disease targets. Created in BioRender. Schank, M. (2026) https://biorender.com/04hpvdb (accessed on 13 February 2026).

Broadly, therapeutic applications of EVs can be categorized into three major domains as illustrated in Figure 5 [29,104,105,106,107,108,109,110,111]: (1) direct treatment, where EVs exert native therapeutic effects; (2) cargo-based therapy in which EVs are engineered to deliver specific therapeutic agents (such as RNA, proteins, or small molecules); and (3) diagnostics where EVs function as non-invasive biomarkers for disease detection and monitoring. Together, these applications establish and place EVs as a promising, multifaceted platform for next-generation therapeutics and personalized medicine.

(1) Direct therapeutic applications of EVs

EVs have shown significant promise as direct therapeutic agents, particularly through their innate biological cargos and capacity for targeted intercellular communication. Examples include antimicrobial activity, immune modulation, tissue regeneration, and cancer immunotherapy (Figure 5). One study explored a universal immunotherapeutic strategy for hepatocellular carcinoma (HCC) using EV-based vaccines [100,112]. This study focused on DC-derived EVs engineered with an HCC targeting peptide, α-fetoprotein epitope, and a functional domain of high mobility group nucleosome-binding protein 1 (N1ND-N). These EVs were shown to elicit robust tumor-specific immune responses in mice with orthotopic HCC, including the activation of both adaptive and innate immune responses. Notably, they also induced long-lived protective T-cell memory, demonstrating the potential of EV-based therapies to provide durable immune protection against HCC [112]. Engineered exosomes have also been shown to deliver therapeutic cargo in vivo, including antitumor microRNAs targeting EGFR in breast cancer tissues [113]. MSC EVs have also been shown to protect kidneys from acute tubular injury in mouse models [114]. Protein-lipid interactions have also been shown to direct protein association with EV membranes, thereby improving loading efficiency and functional delivery of EV cargos [115].

In cardiovascular research, MSC-derived EVs were utilized as an in vivo treatment for mice undergoing myocardial ischemia/reperfusion (I/R injury) [116]. Specifically, mice underwent 30 min ischemia followed by reperfusion, which induced impaired ATP production, elevated oxidative stress, and cell apoptosis. MSC-derived EVs were administered 5 min before reperfusion. This study demonstrated that EV treatment reduced infarct size in a dose-dependent manner by 45% compared to saline treatment via direct EV-cardiac cell interactions; however, this recovery was abolished when EV structural integrity was disrupted by agitation in a homogenizer. Furthermore, the MSC-derived EVs increased ATP levels, alleviated oxidative stress, and activated pro-survival signaling pathways (PI3K/Akt) within an hour of reperfusion during I/R injury and promoted long-term (28-day follow-up) myocardial function and geometry recovery. These results suggest a potential for MSC-derived EVs to improve cardiac function and structure following I/R injury [116]. Although these EVs were not loaded with specific cargo, this study demonstrated that their therapeutic effects may arise from naturally occurring contents. Additionally, the intact EV structure appears crucial for efficient cellular uptake and intracellular delivery and signaling, highlighting the importance of structural integrity for their therapeutic potential.

EVs have also been widely explored in wound healing. A meta-analysis of adipose stem cell (ADSC)-derived EVs in animal wound models, including diabetic ulcer wounds, found that EV treatment significantly enhanced wound healing processes, including neovascularization, epithelialization, collagen fiber organization, and reduced scar formation [117]. In a diabetic mouse model, treatment with human ADSC-derived EVs accelerated wound closure, promoted angiogenesis and collagen synthesis, improved skin barrier repair, and reduced inflammation [118]. Similar regenerative effects, including decreased reactive oxygen species and modulation of keratinocytes and fibroblasts, were observed with EVs derived from bone marrow stromal cells and other mesenchymal stem cells [119]. Mechanistically, these wound-healing effects appear to be mediated in part by exosomal regulatory RNAs, such as circular RNAs (circRNAs), long non-coding RNAs (lncRNAs), and microRNAs (miRNAs), which influence key pathways in tissue repair and regeneration [120,121,122]. Collectively, these studies highlight the diverse and potent therapeutic effects of EVs when used directly, leveraging their intrinsic biological activity and signaling capacity across a broad range of disease contexts.

(2) Cargo-based therapeutic applications of EVs

EVs have emerged as promising drug-delivery vehicles due to their inherent capacity to encapsulate and transport diverse molecular cargos, including proteins, RNAs, lipids, and even gene-editing tools, to recipient cells. Leveraging their natural role in intercellular communication, EVs can be engineered to deliver therapeutic agents with high specificity and minimal toxicity. Their endogenous origin imparts several advantages over synthetic delivery systems, including improved biocompatibility, reduced immunogenicity, and the ability to bypass physiological barriers such as the blood–brain barrier (BBB). These features make EVs particularly attractive for treating conditions where conventional therapies are limited by off-target effects, poor tissue penetration, or immune clearance [123]. Examples of cargo-based therapeutic applications of EVs include gene therapy for the delivery of CRISPR or RNAs (mRNA, miRNA, lncRNA, siRNA, shRNA, etc.) and drug-loaded EVs (Figure 5). For example, in vivo biodistribution and functional delivery of nucleic acids, including plasmid DNA (pDNA), mRNA, and siRNA into EVs has been documented in rodents [124,125].

EVs can also be used as targeted delivery vehicles by altering the expression of EV surface proteins or peptides (for cell and tissue specificity) and internal cargos (for drug target specificity). For example, cancer-focused EV treatment has also utilized EVs as a delivery vehicle for small interfering RNAs (siRNAs). A study targeting glioblastoma (GBM) utilized EVs modified with a brain-tumor-targeting peptide and loaded with small interfering RNA (siRNA) against programmed death-ligand 1 (PD-L1) [126]. These EVs successfully crossed the BBB, reversed radiation-induced PD-L1 expression in tumor cells, and reprogrammed the tumor microenvironment by recruiting myeloid cells and activating CD8^+^ T cells. This approach enhanced immune checkpoint blockade and demonstrated a synergistic therapeutic effect when combined with radiation therapy.

Another study applied a multiplex RNA interference strategy to overcome resistance to Sorafenib in hepatocellular carcinoma (HCC). Researchers engineered HEK293T cell-derived EVs to deliver multi-siRNAs targeting two key regulators of ferroptosis suppression, GPX4 and DHODH. These EVs were equipped with a designer fusion protein comprising: (i) the RNA recognition motif (RRM) of U1-A to bind the multi-siRNA construct, (ii) Lamp2b for exosomal membrane integration, and (iii) the SP94 peptide for HCC cell targeting. The resulting EVs demonstrated high siRNA loading efficiency, selective tumor cell binding, and restored Sorafenib sensitivity by reactivating ferroptosis in both in vitro and in vivo HCC models [127]. These results underscore the feasibility of EV-based multiplex RNA delivery to overcome therapeutic resistance.

EVs have also been utilized for alternative gene-editing applications, particularly for delivering CRISPR/Cas9 ribonucleoproteins (RNPs). In one study, EVs derived from hepatic stellate cells (HSCs) were loaded with Cas9 RNPs targeting genes involved in acute liver injury, fibrosis, and hepatocellular carcinoma (e.g., PUMA, CCNE1, and KAT5). The RNPs were encapsulated via electroporation and demonstrated effective delivery and gene editing in vivo, with liver-specific accumulation [128]. This approach highlights the utility of EVs for delivering large macromolecular therapeutics, such as RNPs, which are otherwise difficult to transport by the most commonly used viral and non-viral delivery approaches due to their size and susceptibility to degradation. This study also provides insight into the potential for EVs to be optimized to direct therapeutics to certain cell types by isolating EVs from a compatible cell source (e.g., HSCs) to target (e.g., liver) cells to improve drug targeting and specificity. Additional proteins have been packaged into EVs and shown to maintain biological activity following delivery [129,130].

Recently, studies have also highlighted the ability to encapsulate drug therapies into EVs for improved delivery. For example, one study utilized tumor-derived EVs that were modified to express a tumor-targeting peptide and loaded with Ginsenoside Rb1 (Rb1)—a promising anti-cancer drug for the treatment of non-small cell lung cancer (NSCLC). These tumor-targeting EVs demonstrated in vitro and in vivo suppression of tumor growth and metastasis, as well as specific tumor targeting and enhanced stability and bioavailability of Rb1 [131]. Furthermore, another study evaluated EVs as a drug delivery vehicle for breast cancer treatment. EVs derived from mesenchymal stromal cells were transduced with ligands to enhance HER2^+^ specificity and packaged with doxorubicin. These engineered EVs demonstrated preferential uptake by HER2^+^ cells and a significant reduction in cell viability in a dose-dependent manner [132]. Additional studies have demonstrated similar success utilizing EVs as a drug delivery vehicle for packaging a diverse range of drug types for delivery to various cell types [31,133,134,135,136].

Importantly, EV-based therapies can combine both intrinsic and engineered therapeutic mechanisms. A recent study demonstrated this dual approach by developing an engineered, DC-derived, EV-based vaccine for breast cancer [137]. These EVs were surface-modified to express α-lactalbumin (α-LA) and electroporated with immunogenic cell death (ICD) inducers—human neutrophil elastase (ELANE) and Hiltonol (a TLR3 agonist). The resulting EVs induced ICD in breast cancer cells and stimulated strong CD8^+^ T cell responses in immunocompetent mice and patient-derived tumor organoids [137]. This synergistic design integrates direct immune activation with targeted cargo delivery, further enhancing EV therapeutic potential.

In summary, EV-based cargo delivery represents a highly adaptable and effective therapeutic platform. Through surface engineering and the incorporation of diverse payloads, including RNAs, proteins, and gene-editing complexes, EVs can be tailored to specific disease contexts, offering precision treatment strategies with minimal toxicity.

(3) EVs as diagnostic and prognostic biomarkers

In addition to their therapeutic applications, EVs have emerged as promising candidates for non-invasive diagnostics and prognostics due to their ability to encapsulate disease-specific biomolecules reflective of their cell of origin. Circulating EVs contain diverse molecular contents that can serve as biomarkers for a wide range of conditions and pathologies, including cancers, obstetrics, and infectious, autoimmune, inflammatory, neurodegenerative, cardiac, and metabolic diseases (Figure 5). For instance, in GBM, EVs isolated from patient serum were found to carry RNAs and proteins associated with tumor progression and angiogenesis, highlighting their potential as liquid biopsy tools for brain tumor detection [16]. Similarly, in NSCLC, serum-derived EVs were screened for microRNAs (miRNAs), revealing distinct miRNA expression profiles in NSCLC patients compared to healthy controls [138]. These findings demonstrate the utility of exosomal miRNAs in distinguishing malignant from non-malignant states. Beyond oncology, exosomal biomarkers have shown promise in obstetrics. A study investigating pregnant women with chronic hypertension, gestational hypertension, and varying severities of preeclampsia identified differentially expressed miRNAs in serum-derived EVs. The expression patterns correlated with disease severity, suggesting a role for exosomal miRNAs in monitoring maternal hypertensive disorders [139].

Neurodegenerative diseases, such as Alzheimer’s disease (AD), have also been explored using exosomal biomarkers. In a mouse model of AD (utilizing intracerebral streptozotocin injection to induce AD-type neurodegeneration), both brain- and serum-derived EVs contained hallmarks of AD pathology—including markers of myelin loss, neuroinflammation, oxidative stress, and amyloid/tau accumulation—when assessed via a non-invasive liquid biopsy approach [140]. These results support the potential of EVs to reflect central nervous system pathophysiology and offer a window into neurodegenerative processes through peripheral blood sampling. Collectively, these studies underscore the potential of EVs as robust, minimally invasive biomarkers for disease detection, monitoring, and prognosis across a diverse array of conditions [87,106].

## 6. Challenges and Future Directions

Despite the growing interest and rapid progress in EV research, several critical challenges must be addressed to fully translate their clinical potential as therapeutic agents and drug delivery vehicles, including standardization of isolation and characterization methods, cargo loading efficiency and specificity, specific targeting and biodistribution, scalability and manufacturing, and immunogenicity and safety. A major hurdle in the EV field is the lack of standardized protocols for EV isolation, purification, and characterization [141]. Current methods, such as ultracentrifugation, size exclusion chromatography, and precipitation-based isolation, vary in yield, purity, and scalability, which complicates comparisons across studies. Developing approaches and reproducible methods for EV production is essential for their clinical applications. MISEV recommends comprehensive reporting of all variables relevant to EV collection, processing, and characterization to promote transparency, reproducibility, and protocol standardization [10]. At minimum, this includes a detailed description of EV source (including donor characteristics for human and non-human samples), cell culture conditions (including but not limited to cell density, viability, vessel system, surface coating, temperature, culture medium, and EV-depletion strategy of media prior to culture), and the quantity and quality of the starting material. Additionally, methodology for sample collection, pre-and post-isolation storage conditions (temperature, duration, freeze–thaw cycles), and appropriate contamination controls should be clearly documented. Additional recommended details include reporting of pre-analytical variables, quantitative input and yield metrics, minimal EV identity criteria using positive and negative markers (using a five-component framework), assessment of co-isolated components, and appropriate controls for functional assays. Inclusion of an explicit isolation and characterization workflow to clarify experimental rationale, technical limitations, and details of downstream analyses is beneficial. Furthermore, while engineered EVs offer promising capabilities for targeted cargo delivery, current methods for loading RNA, proteins, or small molecules, including electroporation, transfection, or passive incubation, often suffer from low efficiency, poor cargo stability, or unintended structural alterations [133]. New strategies that enhance loading precision while preserving EV integrity are needed to improve therapeutic potential.

Achieving cell-specific or tissue-specific delivery remains a significant challenge. Although surface modifications with targeting peptides or ligands (e.g., SP94 for hepatocellular carcinoma) have shown promise, off-target accumulation, especially in the liver and spleen, is still common. More sophisticated engineering approaches are required to improve targeting specificity and minimize systemic clearance. Additionally, translating EV-based therapies from research to the clinic will require scalable manufacturing processes that maintain EV quality and function. Producing clinical-grade EVs under good manufacturing practice (GMP) conditions remains difficult due to batch variability and low yield. Due to the novelty of the field, there is currently no dedicated set of regulatory requirements specific to EV-based therapies. Nevertheless, multiple variables must be carefully controlled to ensure safety, quality, and successful translation to clinical practice. These include, but are not limited to, standardized confirmation of EV identity (particle size, positive marker expression, morphology) and purity (negative markers), assessment of sterility and endotoxin or mycoplasma contamination, implementation of appropriate potency assays, and definition of acceptable batch-to-batch variability [10,95,142]. In parallel, strategies to control EV biodistribution and engineer uptake beyond primary hepatic accumulation are critical, particularly given the tendency of systemically administered EVs to localize to the liver. These GMP and quality-control considerations define a starting point for translating EVs into the clinic.

To unlock the full therapeutic potential of EVs, future research should focus on (1) utilizing optimizable synthetic nanotechnology with EVs to create programmable, modular EVs with tunable properties and combine the advantages of natural vesicles with the scalability of synthetic nanoparticles [143]; (2) exploring combination therapies where EVs are used alongside conventional drugs, gene editing tools (e.g., CRISPR/Cas9), or immunotherapies; and (3) advancing regulatory frameworks that address the unique challenges of EV-based products and streamline clinical translation. EV-based therapies represent a novel paradigm in regenerative medicine, oncology, precision drug delivery, immunotherapy, and gene therapy. Their unique capacity to mirror the biological activity of parent cells, combined with their engineering flexibility and relative safety, supports their continued exploration in preclinical and clinical settings. Ongoing clinical trials underscore their promise, although regulatory, manufacturing, and targeting challenges remain to be addressed before widespread clinical applications.

## Figures and Tables

**Table 1:** EV Isolation Techniques.

Isolation Method	Principle	Purity	Yield	Sample Volume	Processing Time/Throughput	EV Integrity	Specificity	Cost per Sample	Advantages
Ultracentrifugation (UC)	Sedimentation by centrifugal force	Low to moderate	High	High	Long/Low	Moderate	Low	Low	Standardized, no chemicals, relatively high yield
Density Gradient Centrifugation	Separation by buoyant density	High	Moderate	High	Long/Low	High	Moderate	Moderate	High purity, separates vesicle subtypes, preserves activity
Tangential Flow Filtration (TFF)	Filtration by size with tangential flow	Moderate to high	High	Moderate	Moderate/Moderate	High	Low	Low	Scalable, gentle
Size Exclusion Chromatography (SEC)	Separation by size through porous beads	High	Moderate	Moderate	Short/Moderate	High	Low	Low	High purity, preserves structure, and reproducible
Immunoaffinity Capture	Binding to specific surface markers	Very high	Low to moderate	Moderate	Moderate/Moderate	High	Very high	Moderate	High specificity, isolates subpopulations, useful for diagnostics
EXODUS Technology	Size-based microfluidic filtration	High	High	Low-moderate	Short/High	High	Moderate	Moderate	No chemicals, preserves activity, scalable
Membrane-affinity Capture (QIAGEN ExoEasy)	Proprietary membrane binding of vesicles during buffer treatment	Low	Moderate	Moderate	Short/High	High	Low	Moderate	Scalable, good RNA yield

**Table 2:** EV Characterization Techniques.

Technique	Principle	Information Provided	Advantages	Limitations
Transmission Electron Microscopy (TEM)	Transmission of electrons through thin sample	Internal structure Morphology Size	High resolution (1–2 nm)	Complex sample preparation Altered native morphology Low throughput
Scanning Electron Microscopy (SEM)	Surface imaging via electron scattering	Surface morphology	High resolution (1–10 nm)	No internal structure Altered native morphology
Cryogenic Electron Microscopy (Cryo-EM)	Imaging frozen hydrated samples	Morphology Size Internal structure Membrane integrity	High resolution (0.3–1 nm) Visualize EV ultrastructure Preserves native structure	Expensive Technical expertise required Low throughput
Atomic Force Microscopy (AFM)	Surface scanning	Surface topography Stiffness Elasticity Adhesion	Label and stain-free 3D profile Non-destructive Near native state	Low throughput No information on internal structure Technical expertise required
Western Blotting	Protein detection	Protein content Marker presence	Marker verification Low cost	Requires a purified protein sample Does not assess EV size, concentration, or structure
Flow Cytometry (FCM)	Light scattering & fluorescence	Surface markers Phenotyping Approximate concentration	Multiplexing High throughput Quantitative (with optimization)	Requires a high-resolution or nano-flow cytometer Requires labeling No morphology or structural details
Dynamic Light Scattering (DLS) *	Brownian motion detected by light scattering	Size distribution (intensity-based)	Quick Easy to use Low cost	Sensitive to contaminants Biased to large particles No concentration information
Nanoparticle Tracking Analysis (NTA) *	Brownian motion of individual particles	Size distribution Concentration	Real-time tracking Quantitative Single-particle resolution Fluorescence mode	Lower resolution for polydisperse or small EVs (<50 nm)

* NTA is preferred over DLS for heterogeneous EV populations, whereas DLS is limited to relatively monodisperse samples and qualitative size assessment.

## Data Availability

Data sharing is not applicable. No new data were created or analyzed in this study.
